# Short-Term Curative Effect of Endovascular Stent-Graft Treatment for Aortic Diseases in China: A Systematic Review

**DOI:** 10.1371/journal.pone.0071012

**Published:** 2013-08-12

**Authors:** Siwen Wang, Jinsong Wang, Peiliang Lin, Zhibin Li, Chen Yao, Guangqi Chang, Xiaoxi Li, Shenming Wang

**Affiliations:** 1 Department of Vascular and Thyroid Surgery, the First Affiliated Hospital of Sun Yat-sen University, Guangzhou City, China; 2 Department of Epidemiology, the First Affiliated Hospital of Sun Yat-sen University, Guangzhou City, China; The University of Tennessee Health Science Center, United States of America

## Abstract

**Introduction:**

We analyzed the short-term efficacy of endovascular treatment for aortic diseases by summarizing all available published data on endovascular stent-graft treatment for abdominal aortic aneurysm (AAA), thoracic aortic aneurysm (TAA), type A aortic dissection (type A AD) and type B aortic dissection (type B AD) in China.

**Methods:**

We performed a systematic analysis of 935 published series on retrograde endovascular treatment for aortic diseases in China from January 1996 to November 2010. Based on the inclusion criteria, 159 studies, involving a total of 5531 patients, were included.

**Results:**

There were no significant differences in procedural success among the studies (P>0.05). The rates of overall neurologic complications and stroke were significantly different in all two-group comparisons (P<0.01). The type A AD patients had the highest rates of neurologic complications (both 6.67±0.00%), and the AAA patients had the lowest rates (0.31±0.04% and 0.11±0.02%). Significant differences were noted in the rates of cardiac, renal, pulmonary and visceral complications, which were all higher in the type A AD patients than in the other three groups (P<0.01). The endoleak rate was highest in the TAA patients (19.27±5.74%) and was similar in the type A AD patients (P>0.05). A significant difference was noted between the 30-day mortality rate of the type A AD patients and the AAA or type B AD patients (P<0.05).

**Conclusion:**

Endovascular stent-graft is a feasible and safe treatment for aortic diseases, with high procedural success and low incidences of post-procedural complications and short-term mortality. Endovascular treatment for AAA and type B AD is more efficient than for type A AD and TAA.

## Introduction

Aortic diseases derived from injury, degeneration or congenital deformation, including aortic aneurysm (AA) and aortic dissection (AD), endanger the lives of patients. The traditional treatment of true AA and AD is open surgery, which has definite curative effects [1]. Endovascular stent-graft treatment was developed in the 1990s. In 1991, Parodi et al. [2] first reported the application of an endovascular repair technique for the treatment of abdominal aortic aneurysm (AAA). In 1994, Dake et al. [3] used transluminal stent-graft placement to treat thoracic aortic aneurysm (TAA). In 1999, Dake et al. [4] and Nienaber et al. [5] separately reported that endovascular stent-graft placement was successfully used to treat Stanford type B aortic dissection (type B AD). In addition, Sueda et al. [6] and Mizunoa et al. [7] developed stented elephant-trunk transplantation procedures for the endovascular treatment of Stanford type A aortic dissections in 1999 and 2002, respectively (type A AD). In 2002, Kato et al. [8] developed another new method, total arch graft implantation with open-style stent-graft placement. In addition, in China, Jing et al. [9] reported endovascular graft exclusion applied to the treatment of AAA in 1998. In the next year, Wang et al. [10] and Jing et al. [11] separately reported the earliest endovascular stent-graft placements in type B AD patients in China. In 2002, Sun et al. [12] reported the application of the stented elephant trunk procedure in type A AD treatment. Currently, endovascular treatment is widely applied to treat large-artery diseases and is being performed in many medical centers in China. In this review, based on the pre-defined inclusion criteria, we have attempted to summarize all published studies conducted in China (not including Hong Kong, Macao and Taiwan) for endovascular treatment of patients with aortic diseases, including the evaluation of patient characteristics, clinical success, complications and outcomes. Finally, we analyzed and compared the short-term effects of endovascular treatment between AAA, TAA, type A AD and type B AD patients. Based on our comprehensive analysis, we conclude that endovascular stent-graft is a feasible and safe treatment for these aortic diseases in Chinese patients.

## Materials and Methods

### Data Sources

*Aortic dissection*, *stent* and *endovascular* or *transluminal* were the keywords used in our search of the PUBMED, MEDLINE, CBMdisc (Chinese Biomedical Database) and CNKI (Chinese National Knowledge Infrastructure) databases for articles in the English and Chinese literature on the endovascular treatment of aortic dissection involving case studies performed in China with Chinese patients from January 1996 to November 2010. *Aortic aneurysm*, *stent* and *endovascular* or *transluminal* were the keywords used in our search for articles on endovascular treatment for aortic aneurysm from January 1999 to November 2010.

**Figure 1:**
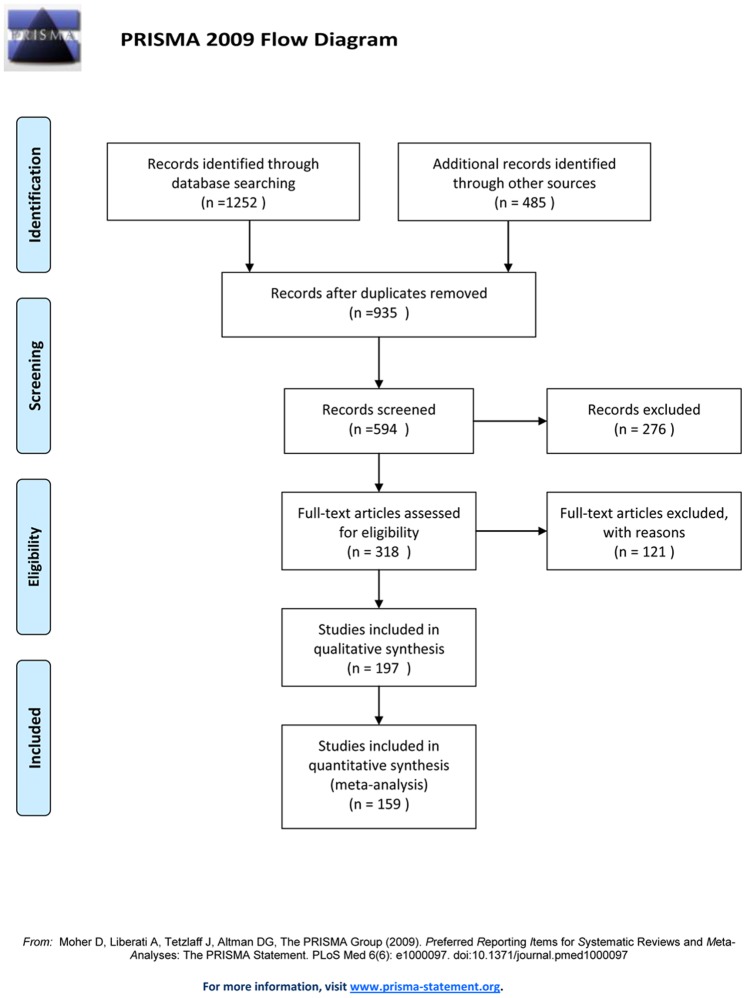
Procedure of articles analysis.

Several criteria were applied to determine whether articles would qualify for analysis: (i) articles including patients with AAA or TAA or aortic dissection (type A or type B); (ii) articles about AAA and TAA with cases ≥2, articles about type A aortic dissection with cases ≥2, and articles about type B aortic dissection with cases ≥6; (iii) if the same center reported a series of cases repeatedly, we selected the newest and most detailed article that included the most cases and the most variables, especially 30-day perioperative data; (iv) if the same center reported different cases in different years, we integrated all cases; (v) articles demonstrating sufficient data; (vi) if an article included one or more types of aortic diseases, we analyzed that article separately; and (vii) if the articles could not be clearly classified into the four types of aortic diseases or if the articles did not present most data, we did not include them for analysis (Figure 1).

### Definitions

This review is on aortic diseases, including AD, AAA and TAA. AD was classified into type A and type B AD according to the Stanford classification system. Pulmonary diseases included COPD (chronic obstructive pulmonary disease), pulmonary emphysema, pulmonary embolism and pneumonia. Renal diseases included disorders of renal function, renal failure, kidney infarction, nephritis and renal calculus. Cerebral vessel diseases included stroke, TIA (transient ischemic attack) and cerebral hemorrhage. Procedural success was defined as procedures in which the stent was anchored in the target location in the aortic lumen. Endoleaks occurring after the first completion of the procedure were reported as outcome data. Procedure-related complications included graft response syndrome, hoarseness, surgical wound infection or injury and other postoperative complications. Overall neurologic complications consisted of stroke (cerebral, cerebellar or brain stem infarction) and ischemia of the spinal cord resulting in numbness and asthenia of limbs. Cardiac complications included myocardial ischemia, myocardial infarction, pericardial tamponade, arrhythmia, heart failure and cardiac arrest. Renal complications involved disorders of renal function, renal failure, renal ischemia and kidney infarction. Pulmonary complications involved pulmonary embolism, pneumonia, respiratory failure, COPD and pulmonary atelectasis. Visceral complications included bleeding ulcer, intestinal ischemic necrosis, intestinal obstruction, intestinal perforation and unknown abdominal pain.

### Data Extraction

Two standardized protocols were used for data extraction: one for articles referring to aortic dissection as introduced by Eggebrecht et al. [13] and one for articles referring to abdominal aortic aneurysm as introduced by Lovegrove et al. [14]. Considering all four types of aortic diseases, a modified standardized protocol, which included pre-defined variables regarding clinical characteristics, procedural data, perioperative (30 days) complications and survival data, was used to analyze each article in this review. The authors independently performed data extraction. Consensus was achieved with further discussion when discrepancies occurred. Unspecified information was classified as not available so articles without these data could not be included when analyzing a specific variable; thus the patient numbers (denominator) varied with the specific variables.

### Statistical Analysis

The standardized protocol used for statistical analysis followed the method introduced by Eggebrecht et al. [13]. The rates of events were calculated as the number of events divided by the number of treated patients with available data. The results are presented as the means ± standard deviations or medians and ranges, as appropriate. Comparisons between the patients with different aortic diseases were made using a two-sided χ^2^ test for categorical variables and a two-sided Student’s t-test for continuous variables. A P-value of <0.05 was considered statistically significant. The Kaplan-Meier non-parametric method was used to estimate mortality, which was compared using the log-rank test. The statistical software SPSS 17.0 (SPSS, Chicago, IL, USA) was used for all statistical analyses.

## Results

### Overview of Studies

We analyzed 935 published series on retrograde endovascular treatment for aortic diseases in China. Based on the inclusion criteria, 159 studies involving a total of 5531 patients were included to compare short-term curative effects. Among these studies, 36 studies (1105 patients) were about AAA, 10 studies (113 patients) were about TAA, 19 studies (343 patients) were about type A AD, and 94 studies (3770 patients) were about type B AD. All clinical characteristics, procedural data and perioperative data are illustrated in Tables 1, 2 and 3.

**Table 1:** Characteristics of patients with aortic diseases.

Variables	AAA (F1, %)	TAA (F2, %)	Type A AD (F3, %)	Type B AD (F4, %)	P_Total_
Mean age (years)	70.85	64.54	47.95	53.37	
Male ratio	89.21±8.08 (843/945)	56.34±13.51 (40/71)	80.52±2.88 (186/231)	84.97±1.19 (2545/2995)	0.000<0.01
Smoker	55.68±9.19 (103/185)	–	42.86±14.14 (15/35)	54.46±5.79 (183/336)	0.371
Hypertension	59.83±4.57 (557/931)	75.00±20.15 (42/56)	67.57±2.76 (150/222)	85.74±1.38 (2922/3408)	0.000<0.01
Coronary heart disease	27.36±2.24 (255/932)	16.67±6.12 (9/54)	17.24±3.59 (10/58)	13.64±0.60 (269/1972)	0.000<0.01
Diabetes	10.78±1.14 (80/742)	16.67±6.12 (9/54)	9.46±2.03 (7/74)	14.17±0.92 (236/1666)	0.093
Pulmonary disease	15.71±1.55 (123/783)	11.11±3.55 (6/54)	–	6.60±0.29 (88/1334)	0.000<0.01
Renal disease	6.68±0.50 (25/374)	7.41±2.14 (4/54)	10±1.67 (6/60)	7.75±0.25 (165/2130)	0.807
Cerebral vessel disease	9.06±0.66 (31/342)	–	8.62±1.22 (5/58)	4.19±0.29 (58/1384)	0.002<0.01
Rupture	1.27±0.57 (1/79)	4.65±0.00 (2/43)	8.00±3.06 (4/50)	8.93±1.92 (31/347)	0.043<0.05

Male ratio: F1>F2 (P = 0.000); F1>F3 (P = 0.000); F1>F4 (P = 0.001); F2<F3 (P = 0.000); F2<F4 (P = 0.000); F3 vs. F4: no significant differences. Hypertension: F1<F2 (P = 0.024); F1<F3 (P = 0.033); F1<F4 (P = 0.000); F2<F4 (P = 0.023); F3<F4 (P = 0.000); F2 vs. F3: no significant differences. Coronary heart diseases: F1>F4 (P = 0.000); No significant differences in other two-group comparisons. Pulmonary diseases: F1>F4 (P = 0.000); No significant differences in other two-group comparisons. Cerebral vessel diseases: F1>F4 (P = 0.000); No significant differences in other two-group comparisons. Rupture: F1<F4 (P = 0.020); No significant differences in other two-group comparisons. (“–” stands for no available data for specific variables in the literature).

**Table 2:** Procedural data of patients with aortic diseases.

Variable	AAA (F1, %)	TAA (F2, %)	Type A AD (F3, %)	Type B AD (F4, %)	P_total_
Procedural success	99.73±6.57 (1102/1105)	98.23±11.23 (111/113)	100±15.35 (78/78)	99.56±1.40 (3753/3770)	0.127
Elective procedures	98.85±11.57 (774/783)	68.52±20.48 (37/54)	100±13.54 (47/47)	86.80±11.54 (296/341)	0.000<0.01
Emergency procedures	1.15±0.22 (9/783)	31.48±18.18 (17/54)	0 (0/47)	13.20±2.25 (45/341)	0.000<0.01
General anesthesia	55.84±4.97 (220/394)	60.47±36.18 (26/43)	100 (25/25)	79.50±1.93 (1268/1595)	0.000<0.01
Need for emergent surgicalconversion	0.29±0.03 (3/1025)	0 (0/113)	0 (0/78)	0.05±0.04 (2/3601)	0.293

Elective procedure:F1>F2 (P = 0.000); F1>F4 (P = 0.000); F2<F3 (P = 0.000); F3>F4 (P = 0.000); No significant differences in other two-group comparisons. Emergency procedures: F1<F2 (P = 0.000); F1<F4 (P = 0.000); F2>F3 (P = 0.000); F3<F4 (P = 0.000); No significant differences in other two-group comparisons. General anesthesia: F1<F3 (P = 0.000); F1<F4 (P = 0.000); F2<F3 (P = 0.000); F2<F4 (P = 0.002); F3>F4 (P = 0.011); F1 vs. F2: No significant differences.

**Table 3:** 30-day perioperative data of patients with aortic diseases.

Variable	AAA (F1, %)	TAA (F2, %)	Type A AD (F3, %)	Type B AD (F4, %)	P_total_
Procedure-related complications	6.41±0.49 (64/999)	2.44±1.09 (2/82)	4 (1/25)	13.96±0.81 (255/1826)	0.000<0.01
Overall neurologic complications	0.31±0.04 (3/980)	4.50±0.91 (5/111)	6.67±0.00 (2/30)	1.66±0.03 (53/3202)	0.000<0.01
Stroke	0.11±0.02 (1/938)	1.96±0.43 (2/102)	6.67±0.00 (2/30)	0.82±0.02 (25/3052)	0.002<0.01
Paraplegia	0.20±0.03 (2/1021)	0 (0/113)	0 (0/47)	0.06±0.01 (2/3540)	0.630
Post-procedural endoleak	10.08±0.52 (110/1091)	19.27±5.74 (21/109)	19.15±1.50 (9/47)	9.39±0.25 (342/3642)	0.004<0.01
Cardiac complications	0.95±0.09 (9/948)	1.22±0.55 (1/82)	10.34±2.44 (3/29)	1.76±0.14 (16/908)	0.025<0.05
Renal complications	1.12±0.17 (10/893)	2.44±1.09 (2/82)	13.79±4.88 (4/29)	2.91±0.15 (43/1476)	0.001<0.01
Pulmonary complications	0.77±0.08 (7/906)	0 (0/82)	10.34±2.44 (3/29)	1.64±0.12 (15/916)	0.005<0.01
Visceral complications	0.92±0.10 (8/868)	0 (0/82)	10.34±2.44 (3/29)	2.78±0.43 (22/791)	0.001<0.01
30-day mortality	2.44±0.20 (27/1105)	3.54±0.62 (4/113)	7.69±1.67 (6/78)	2.36±0.04 (89/3770)	0.023*<0.05
Aorta-related mortality	0.47±0.04 (5/1062)	0.98±0.33 (1/102)	1.28±0.02 (1/78)	0.83±0.02 (27/3269)	0.607
Non-aorta related mortality	1.89±0.20 (20/1062)	2.94±0.69 (3/102)	6.41±1.57 (5/78)	1.53±0.04 (50/3259)	0.008<0.01

Procedure-related complications: F2<F4 (P = 0.003); No significant differences in other two-group comparisons. Overall neurologic complications: F1<F2 (P = 0.000); F1<F3 (P = 0.008); F1<F4 (P = 0.001); No significant differences in other two-group comparisons. Stroke: F1<F2 (P = 0.027); F1<F3 (P = 0.003); F1<F4 (P = 0.018); F3>F4 (P = 0.028); No significant differences in other two-group comparisons. Post-procedural endoleak: F1<F2 (P = 0.003); F1<F3 (P = 0.047); F2>F4 (P = 0.001); F3>F4 (P = 0.044); No significant differences in other two-group comparisons. Cardiac complications: F1<F3 (P = 0.000); F2>F3 (P = 0.000); F3>F4 (P = 0.019); No significant differences in other two-group comparisons. Renal complications: F1<F3 (P = 0.000); F1<F4 (P = 0.004); F2<F3 (P = 0.005); F3>F4 (P = 0.011); No significant differences in other two-group comparisons. Pulmonary complications: F1<F3 (P = 0.000); F2<F3 (P = 0.016); F3>F4 (P = 0.016); No significant differences in other two-group comparisons. Visceral complications: F1<F3 (P = 0.000); F2<F3 (P = 0.016); No significant differences in other two-group comparisons. 30-day mortality: F1<F3 (P = 0.018); F3>F4 (P = 0.003); No significant differences in other two-group comparisons. Aorta-related mortality: F1<F3 (P = 0.025); F3>F4 (P = 0.004); No significant differences in other two-group comparisons (*Log rank test).

### Patient Characteristics (Table 1)

The mean age of the AAA patients was the highest (70.85 years) among all groups, and the mean age of the type A AD patients was the lowest (47.85 years). No significant differences were identified between the male-to-female ratios of the type A AD and type B AD patients (P>0.05), but there were significant differences in the other two-group comparisons (P<0.01). No significant differences were identified in the incidence of hypertension between the type A AD patients and TAA patients (P>0.05), but significant differences were noted for the other two-group comparisons (P<0.01). The incidence of coronary heart disease in the AAA patients (27.36±2.24%) was higher than that in the other groups (P<0.01). The percentages of patients with preoperative aneurysm rupture were similar between the type A AD and type B AD patients (P>0.05). However, there were significant differences when these two groups were compared with the other two groups separately (P<0.01). The type B AD group had the lowest rate of cerebral vessel diseases, which was significantly different from the rates of the AAA and type A AD groups (P<0.01). However, no significant differences in the rates of cerebral vessel disease were noted between the AAA and type A AD groups (P>0.05). There were also no significant differences identified for smoking rates and incidences of diabetes and renal diseases among the four patient groups (Table 1).

### Procedural Data (Table 2)

No significant differences were identified between the aortic disease groups in terms of procedural success (P>0.05). However, significant differences were identified among these four groups in the rates of elective procedures and emergency procedures and in the rates of general anesthesia (P<0.05). The elective procedure rates were all higher and the rates of emergency procedures were all lower in the AAA and type A AD groups than those in the other groups (P<0.01). The type A AD group had the highest rate of general anesthesia (100%), and it was significantly different from those in the other three groups (P<0.01). No significant differences were identified for the rates of emergent surgical conversion among these four groups (P>0.05).

### Perioperative Data (Table 3)

The highest procedure-related complications rate was identified in the type B AD patients (13.96±0.81%), and it was significantly different from that in the TAA patients (p<0.01). However, no significant differences were found for the other two-group comparisons (P>0.05). Except for the procedure-related complications, paraplegia and endoleak, the type A AD group had the highest incidence of every type of 30-day post-operative complication compared with the other three groups (P<0.01). The rate of overall neurologic complications was significantly different in all two-group comparisons (P<0.01), and the AAA patients had the lowest rate (0.31±0.04%). Stroke incidence was significantly different in all two-group comparisons (P<0.01), except for the comparison between the TAA and type B AD groups, which had similar incidences (P>0.05). The incidences of paraplegia were low among the four groups and similar between groups (P>0.05). No cases of paraplegia were observed in the TAA and type A AD groups. Significant differences were noted between groups for cardiac, renal, pulmonary and visceral complications. The rates of these complications were higher in the type A AD patients than those in the other three groups (P<0.01). However, except for the type B AD patients, who had higher numbers of renal complications than did the AAA patients (P<0.01), the other three groups had similar results for these complications (P>0.05). The post-procedural endoleak rate was the highest for the TAA patients (19.27±5.74%) and was similar to that for the type A AD patients (P>0.05). Significant differences were identified for post-procedural endoleak rates between these two groups and the other groups (P<0.01). Significant differences in 30-day mortality were also noted among the type A AD, AAA and type B AD (P<0.05) patients, but no significant differences were found in the other two-group comparisons (P>0.05). No significant differences in aorta-related mortality were noted among the four groups (P>0.05). The type A AD group had the highest non-aorta-related mortality, and it was significantly different from that of the AAA group and type B AD group (P<0.05), but there were no significant differences in the other two-group comparisons (P>0.05).

## Discussion

Although operative technique, anesthesia and pharmacologic therapy have been greatly developed during the past few decades, patients receiving open surgical treatment still face tremendous risks related to intra-operative or post-operative complications. To avoid these disadvantages of open operations, minimally invasive endovascular treatments have been developed for large-artery diseases and have been applied worldwide since the first endovascular aneurysm repair (EVAR) was applied to treat AAA in 1991. Endovascular treatment in large-artery diseases has displayed good short-term and mid-term outcomes. Lovegrove et al. [14] conducted a meta-analysis to compare outcomes after open surgery and EVAR for treating AAA and demonstrated that the incidences of post-procedural complications, 30-day mortality and long-term mortality were lower in the endovascular group. Other international clinical case-control studies have also reported similar results [15]–[17].

According to our results and patient characteristics, the mean age was highest for the AAA patients among the four groups in this study; the patients with type A AD had the lowest mean age. Greenhalgh et al. [16] reported a mean age of 74.0 years for patients treated with EVAR, and Ius et al. [18] reported a mean age of 64 years for patients with type A AD receiving endovascular treatment. All of these results suggest that the mean ages of AAA and TAA patients are higher and that endovascular treatment for true aneurysms is a more mature technique with an easy operative procedure and low risks, allowing it to be applied even to older adults (aged >65 years); however, the type A AD lesion is complicated, and stent implantation is difficult. Therefore, endovascular treatment for type A AD involves longer operative times and has been applied primarily to younger patients (mean age 47.95 years old) in China.

In China in our study, more than 50% of the patients with aortic diseases had hypertension; the incidence was highest in the type B AD patients. The incidence of coronary heart disease was highest in the AAA group, but the incidence of cerebral vessel disease was lowest in the type B AD group. Marin et al. [19] reported the statistical results of 817 patients with AAA and TAA, and the incidence of preoperative complications was as follows: 75% with hypertension, 67% with coronary heart diseases, 13% with cerebral vessel diseases, 25% with renal diseases, 13% with diabetes and 17% with pulmonary diseases; additionally, 47% were smokers. These results suggest that the preoperative complications of aortic aneurysm patients from international studies are similar to those reported for Chinese patients. Zipfel et al. [20] analyzed and summarized the incidences of preoperative complications in type A AD and type B AD patients as follows: 85% with hypertension, 36% with coronary heart diseases, and 31% with pulmonary diseases; additionally, 43% were smokers. Compared with the data from Chinese patients, the incidences of hypertension and smoking were similar, but the incidences of coronary heart diseases and pulmonary diseases were higher in the Zipfel study [20].

The procedural success rates in the four aortic disease groups were all more than 98%, and no significant differences were identified among these four groups in China. The procedural success rates for endovascular treatment reported in international studies [13], [16]–[20] are almost all above 90%. These results indicate that the endovascular technique can be considered a successful treatment for aortic diseases.

Except for procedure-related complications, paraplegia and endoleak, the type A AD group had the highest incidences of 30-day postoperative complications. In an international study [18], the incidences of perioperative complications in patients with type A AD were as follows: 25% with overall neurologic complications, 4% with stroke, 21% with renal complications, 18% with cardiac complications and 4% with visceral complications. In the present study, except for the incidence of stroke, which was similar to international data, and the incidence of visceral complications, which was lower in international study results, the incidences of most other complications were higher in type A AD patients. One explanation for result this is the exceptionally complicated anatomic configuration of the ascending aorta and aortic arch in which the type A AD lesion is located. In addition, the lesion is usually associated with the large vessels branching from the aortic arch. Therefore, these anatomical factors may severely affect the blood supply for critical organs after endovascular treatment, which may lead to a higher incidence of post-operative complications. The incidences of all complications in the AAA patient group, except for endoleak, were the lowest among the four groups reviewed. In an international study, Makaroun et al. [21] reported the incidences of post-operative complications in the AAA group as follows: 8.7% with cardiac complications, 4.7% with renal complications, 4% with vessel complications (including cerebral vessel complications and visceral vessel complications) and 4% with pulmonary complications. That study indicated that endovascular treatment for AAA was the safest. Moreover, because the rate of general anesthesia in the AAA group was the lowest, anesthesia-related complications were correspondingly decreased.

Post-procedural endoleak was still the major complication of endovascular treatment. The rate of post-procedural endoleak was the highest in the TAA patients, and the rate was similar in the type A AD patients. This result can be explained as follows: the anatomic configuration of the aortic arch curves between the ascending aorta and descending aorta, and in this region, the velocity of blood flow is fast, and the blood pressure is high. Consequently, the aortic arch shifts widely along with the heartbeat. In addition, aneurysm is a degenerative process and may accentuate the curving of the aortic arch. Together, these factors can lead to great difficulties in stent anchoring and can result in more frequent post-procedural complications such as endoleak, stent migration and other problems. Therefore, post-procedural endoleak can become the most critical problem for EVAR treatment [22]. The results reported by Leurs et al. [23] indicated that the rate of post-procedural endoleak in TAA patients was 9.2%. Van Marrewijk et al. [24] reported that the rate of post-procedural endoleak in a clinical study of AAA was 6.9%. The rates of endoleak reported in Chinese studies were higher than those from international study data. The Chinese results indicate that endoleak is still the major complication, which requires greater attention from surgeons to be resolved, especially because this endovascular procedure is conducted at most clinical centers to treat aortic diseases.

The 30-day mortality was highest for the type A AD patients in the studies reviewed for this report; the non-aorta-related mortality was also the highest in the type A AD group. Ohki et al. [25] reported a 30-day mortality of 8.5% in AAA patients with endovascular treatment. Leurs et al. [22] reported a 30-day mortality of 10% in TAA cases, of which 28% of deaths were associated with emergency surgery and 5.3% were associated with elective procedures. Ius et al. [18] reported that hospital mortality in type A AD patients was 7%. These results all indicate that type A AD patients have the highest mortality after endovascular treatment, which can be explained as tearing of the ascending aorta or aortic arch in type A AD easily resulting in the rupture of the dissection. In addition, the tearing occurs in the proximal descending aorta, resulting in retrograde tears of the ascending aorta, which may lead to a second intimal tear in the ascending aorta and aortic arch or other severe complications such as pericardial effusion or retrograde rupture.

### Conclusions

Endovascular stent-graft is a feasible and safe treatment with high procedural success, low incidence of post-procedural complications and low short-term mortality. Our results demonstrate that endovascular treatment for AAA and type B AD is more efficient than it is for type A AD and TAA, especially considering that type A AD patients receiving endovascular treatment have higher incidences of post-procedural complications and mortality. Post-procedural endoleak remains the most critical problem related to endovascular treatment, and solutions are urgently needed in China.

## Supporting Information

Checklist S1 (DOC)
